# Necrotizing Fasciitis - Report of ten cases and review of recent literature

**Published:** 2013-06-25

**Authors:** S Al Shukry, J Ommen

**Affiliations:** * Red Crescent Hospital, Almansour, Baghdad, Iraq; ** Rustaq Hospital, Rustaq, Sultanate of Oman

**Keywords:** Cellulitis, Soft tissue infections, Fournier’s gangrene

## Abstract

Necrotizing fasciitis is an uncommon disease that results in gross morbidity and mortality if not diagnosed and treated in its early stages. At onset, however, it is difficult to differentiate from other superficial skin conditions such as cellulitis. Family physicians must have a high level of suspicion and low threshold for surgical referral when confronted with cases of pain, fever, and erythema.

We present ten cases of necrotizing fasciitis managed in a provincial secondary hospital in Oman over 3 years ago. A review of recent literature is also presented.

## Introduction

Necrotizing fasciitis is an uncommon disease that results in gross morbidity and mortality if not treated in its early stages. However, at onset, it is difficult to differentiate from other superficial skin conditions such as cellulitis. Family physicians must have a high level of suspicion and low threshold for surgical referral when confronted with cases of pain, fever, and erythema [**1**].

 Necrotizing Fasciitis (NF) is a rapidly progressive soft tissue infection primarily involving the superficial fascia and subcutaneous tissue. It is caused by Streptococcus pyogens or synergistic infection of aerobic and facultative anaerobic bacteria. NF has been divided into three types based on microbiological cultures. Type I is Polymicrobial and usually caused by a Aerobic and Anaerobic organisms. Type II caused by Streptococci alone or with staphylococci [**2**]. Type III is caused by Marine vibrio [**3**].

## Patients and method 

The computerized records of 10 consecutive patients who have been admitted in our hospital for NF from Jan. 2007 to Dec. 2009 were reviewed retrospectively. This excluded diabetic foot infections, which are well known to the health care providers and considered as Necrotizing cellulites extending deeper in than the skin and subcutaneous tissues.

**Table 1:** Details of the 10 patients with N F

Pt. NO	AGE	SEX	WBC	FEVER	COMORBIDITY	NO OF OP	OP. TIMING	SITE	Hospital admission days
1	70Y	M	23.5 K/ul	+	DM	3	1st day	Perineum & upper thigh Pain, erythema & crepitus	27 days
2	52Y	M	22.2 K/ul	+	DM	5	1st day	Rt. thigh & popliteal fossa. Painful warm red skin	90 days
3	48Y	M	14 K/ul	+	DM	1	10th day	Red Skin- with blisters	80 days
4	53Y	F	13.4 k/ul	-	-	1	2nd day	Right leg Pain, Erythema and swelling	35 days
5	61Y	M	11.8 k/ul	-	CAD & Renal	1	1st day	Anterior abd. Wall Scrotum & perineum severe pain	One day. Died
6	74Y	M	14.8 k/ul	+	-	1	1st day	Fournier’s gang.	One day. Died
7	52Y	M	10.2 K/ul	-	-	1		Scrotum, ant. abdominal wall and left buttock Swelling, discharging pus	30 days
8	44Y	M	17.3 K/ul	-	-	1	1st day	Fournier’s Swelling and redness	33 days
9	31Y	M	12.7 K/ul	+	-	2	1st day	Fournier’s Swelling and redness	28 days
10	54	M	23.3 K/ul	+	DM	1	14th day	Left thigh & scrotum Swelling with pus discharge	60days Died

## Results

The results collected for each of the 10 patients were age, gender, pre-disposing factors, presenting signs and symptoms, location of infection, laboratory findings, microbiological cultures, the type of therapy used, treatment outcome and number of days in the hospital. Age ranged from 31 to 74 years (average age53.9 +/_12.37785). No children seen in this group [**3**].There was only one female in this group. The most common associated comorbidity was DM in 6. One patient was on long-term immunosuppressant following renal transplantation. Regarding the site of infection, 6 patients (60%) had Fournier’s gangrene- (NF of the perineum and genital regions). The other 4 cases of NF involved the lower extremities. Severe local pain with swelling and erythema were seen in all patients. Crepitus in 2 cases and Fever in 4 cases were observed on admission. Leucocytosis was observed in 7 cases. Average (WBC16.48889 +/- 4.961955). The organisms isolated; Staph. aureus –in 3 cases. Mixed polymicrobial infection and Enterobacteracea like Klebsiella, E coli and Proteus mirabilis were identified in 3 cases. No growth was seen in 4 patients. Tissue biopsy was confirmatory for Necrotizing fasciitis in 3 cases. Most of our patients received triple antibiotic therapy to start with. Later they were tailored as per culture and sensitivity reports. Many of these patients were subjected to multiple debridements ranging from 1 to 5 surgeries (mean being 2 operations). Skin grafting was undertaken in our hospital for 4 of these patients while two had to be referred to the Plastic surgery unit for major surgical procedures. The number of hospitalization days ranged from 90 days to one day. Average (37.3 +/-26). Mortality in this study was found to be 30%- one patient dying on the day of admission following the extensive debridement.

## Discussion

This condition was described in several reports in the late 1800s, and it was Dr. B. Wilson who first termed the condition Necrotizing fasciitis in 1952 [**4**]. The disease occurs infrequently and can affect any part of the body.

### Pathogenesis

Necrotizing fasciitis is classified according to its microbiology (polymicrobial or monomicrobial), anatomy, and depth of infection. Polymicrobial NF mostly occurs in immunocompromised individuals. Monomicrobial NF is less common and affects healthy individuals who often have a history of trauma (usually minor). Patients with NF can present with symptoms of sepsis, systemic toxicity, or evidence of skin inflammation, with pain that is disproportional to the degree of inflammation. However, these are also present in less serious conditions. Hyperacute cases present with sepsis and quickly progress to multiorgan failure, while subacute cases remain indolent, with festering soft-tissue infection. Because the condition is rare with minimal specific signs, it is often misdiagnosed [**1**]. NF has been divided into two types based on microbiological cultures. Type I is polymicrobial, usually caused by aerobic and anaerobic organisms, and Type II caused by streptococci alone or with staphylococci [**2**]. The destruction of tissues and thrombosis is caused both by the toxins, antigens, the enzymes of Group A Streptococcus, and the host response to the antigens. Virulent mechanisms include cell wall attached proteins, proteases, exotoxins, and super-antigens. T-helper lymphocytes are activated; in turn activating cytokines, clotting factors, and complement factors. Cytokines include tumor necrosis factor, interleukin-1β, interleukin-2, and interferon [**3**]. Howard et al. reported 18 patients in 1985- affected by a specific type of NF caused by Vibrio vulnificus. These results when a person, usually someone with liver function problems (for example alcoholics or immunosuppressed patients) eats contaminated seafood or a wound is contaminated with sea water containing Vibrio vulnificus [**5**]. One case reported due to Vibrio parahaemolyticus [**6**]. Morganella morganii and "diphtheroids [**7**] Mucormycosis is a rare cause of necrotizing fasciitis reported in a caesarean wound in young female patients [**8**]. Also fungal periorbital NF reported in an immunocompetent adult [**9**]. Cryptococcus neoformans reported in a patient with pemphigus vegetans [**10**]. NF has been reported after Laparoscopic appendicectomy [**11**], cholecytectomy [**12**] and after medical termination of pregnancy [**13**]. An unusual case of Necrotizing fasciitis of the thigh due to the spread of sigmoid colon cancer was reported. However, this fatal complication should be considered during chemotherapy for patients with unresectable colorectal cancer [**14**]. One case was reported following self -induced traumatic rectal perforation [**15**].

### Clinical features

**Table 2:** Clinical features suggestive of necrotizing soft tissue infections

SKIN	PAIN	GENERAL
Erythema with ill-defined margins	Pain that extends past margin of apparent infection	Fever with toxic appearance
Tense edema with grayish or brown discharge	Severe pain that appears disproportionate to physical findings	Altered mental state
Lack of lymphangitis or lymphadenopathy	Decreased pain or anesthesia at apparent site of infection	Tachycardia
Vesicles or bullae, hemorrhagic bullae		Tachypnea due to acidosis
Necrosis		Presentation with DKA or HHNK
Crepitus		
:DKA—diabetic ketoacidosis, HHNK—hyperosmolar hyperglycemic non-ketotic acidosis.		

The majority of cases begin with a pre-existing infection, most commonly on an extremity. This portal of entry may be small or trivial, most often remaining undetected. The difficulty in making an early diagnosis is due to the paucity of cutaneous findings in the early course of the disease. Preadmission treatment with antibiotics modified the initial clinical picture and often masked the severity of the underlying infection. The most common associated comorbidity was diabetes mellitus (sixty-three patients; 70.8%). Advanced age, two or more associated comorbidities, and a delay in surgery of more than twenty-four hours adversely affected the outcome. Multivariate analysis showed that only a delay in surgery of more than twenty-four hours was correlated with increased mortality [**16**]. Early recognition and expeditious initial wide excision and debridement along with an appropriate antibiotic coverage and support of systemic effects of necrotizing fasciitis serve to decrease morbidity and mortality [**17**].

Severe local pain and a significantly elevated white cell count on admission should alert the physician to the presence of severe infection and prompt the initiation of expeditious aggressive treatment [**17**]. Early symptoms resemble those of cellulites but progressive skin changes such as skin ulceration, bullae formation, gas formation in the tissues, and fluid draining from the site can occur rapidly as the infection progresses. Hemorrhagic bullae and crepitus are sinister signs, with the likelihood of underlying fascia and muscle being compromised. Two of the patients in our study had crepitus denoting gas formation while bullae formation was noted in only in one. Crepitus is a late sign however, and is found in only about 18% of cases of NF [**1**].

**Table 3:** Risk factors for necrotizing fasciitis

Risk factors for necrotizing fasciitis
• Diabetes
• Chronic disease
• Immunosuppressive drugs (e.g. prednisolone)
• Malnutrition
• Age > 60 years
• Intravenous drug misuse
• Peripheral vascular disease
• Renal failure
• Underlying malignancy
• Obesity

In one review, thirty-three patients were studied over a 3-year period. Predisposing factors (**Table 2**) included intravenous drug abuse (30%), diabetes (21%), and obesity (18%). Severe pain (94%) and abnormal temperature (88%) were present, whereas laboratory data and X-ray were nonspecific. Gram-positive organisms were most frequently recovered (B-hemolytic streptococcus 45%). Treatment consisted of antibiotics, surgical debridement, re-exploration 24 hours before surgery, nutritional support, and early soft tissue coverage as needed. Mean duration from admission to operation was of 43 hours. The average number of operative debridements was of three and the average length of hospitalization was of 47 days. Patients operated on less than 12 hours from admission or greater than 48 hours, had shorter hospital stays (36 and 38 days). The critical time period was 12-48 hours after admission; all deaths and amputations were in this group; were 62 days [**18**].

The sequence of developing the cutaneuos lesions in Twenty-two patients was identified. At initial assessment (day 0), almost all patients presented with erythema, tenderness, warm skin, and swelling. Blistering occurred in 41% of patients at presentation whereas late signs such as skin crepitus, necrosis, and anesthesia were infrequently seen (0-5%). As time elapsed, more patients had blistering (77% had blisters at day 4) and eventually the late signs of necrotizing fasciitis characterized by skin crepitus, necrosis, and anesthesia (9-36%) were seen. A clinical staging system was developed based on our observations. Stage migration from early to late stage necrotizing fasciitis was evident with the majority of patients in stage 1 on day 0 (59%), whereas by day 4, the majority had developed into stage 3 (68%) [**19**].

### Diagnosis

 The disease commonly occurs in patients in the 4th to 7th decades and is more common in men. Severe local pain and a significantly elevated white blood cell count on admission should alert the physician of the presence of severe infection and prompt the initiation of expeditious aggressive treatment [**20**]. The diagnosis of NF in the early stages may be challenging because it can be confused with less serious conditions such as erysipelas and cellulites. However, NF typically presents with pain that is out of proportion to the degree of skin inflammation. However, certain patients especially those with diabetic neuropathy can experience minimal pain, resulting in a missed diagnosis. This is more likely to occur in concealed sites of infection such as perineum or oral cavity. In contrast to cellulites no Lymphangitis or lymphadenitis.

NF can take a hyper acute or a sub acute course of progression. Patients in the former group present with sepsis and rapidly progress to multi-organ failure. The diagnosis of sepsis is obvious in their case. Several authors have described a sub acute variation of NF [21,22], thes e patients have an indolent disease course, with festering soft tissue infection. Once a certain threshold is reached, sudden deterioration is the rule in this group of patients.

Diabetes mellitus is the leading pre-disposing factor in our patient population. The mechanisms that have been suggested for underlying how DM could cause susceptibility to NF are the following:

a) The peripheral sensory neuropathy leading to increased susceptibility to minor trauma

b) Tissue hypoxia caused by diabetic vascular disease and the underlying immunodeficiency [**23**]

 Even though there is substantial evidence indicating an important role of DM in the etiology of NF, its role as a predisposing factor for increased death rate is controversial. Some reports failed to show a significant relationship between mortality and DM in NF [**24**].

 Fournier’s gangrene is an infectious necrotizing fasciitis of the perineum and genital regions. The dominant clinical features of Fournier’s gangrene include general malaise, severe pain and tenderness in the perineum, and ulceration perineum. The patient’s metabolic status and the extent of disease at presentation is an important factor in the prognosis of Fournier’s gangrene [**25**].

 To help decide which patients require a surgical exploration, particularly in those with equivocal clinical signs, laboratory and radiological tests might sometimes be useful. 

### Investigations

 Leucocytosis is one of the indicators in laboratory risk indicator score for NF (LRINEC) for early diagnosis and differentiating NF from other soft tissue infections with more than 90% sensitivity and specificity [**26**]. 

 Blood cultures are usually part of the workup in hospital and might yield up to 27.3% positive cultures in necrotizing infections, compared with the mere 2% positive blood culture yield in patients with cellulitis. 

 Plain X-ray films can demonstrate subcutaneous gas, but this is a specific not a sensitive finding (positive in fewer than 25% cases and absence of gas does not exclude NF [**27**].

 Computed tomography (CT) and magnetic resonance imaging (MRI) might be useful in cases where signs are equivocal and diagnoses in doubt. Asymmetrical fascial thickening, fat stranding, and gas tracking along with fascial planes are important imaging findings. CT scans are estimated to have a sensitivity of 80% for detecting necrotizing soft tissue infections [**28**].

 MRI can detect the extent of NG and it can identify soft tissue edema infiltrating the fascial planes, many hours prior to cutaneous signs of infection or local gangrenous changes allowing rapid diagnosis and treatment and improved outcome [**29**]. 

### Treatment

 The treatment of NF is a combination of surgical debridement, appropriate antibiotics and optimal oxygenation of the infected tissues. The priority in every case is to proceed to radical surgical debridement. Patients should be told about the gravity of their condition and the risk of increased mortality if surgical debridement is not performed.

Broad-spectrum antibiotics effective against the anaerobes, gram negative and gram-positive bacilli are started immediately on admission. In our study group, triple antibiotics were used in the beginning for most of the cases (Consists of Cephredine, Gentamycin and Metronidazole given for a period of 5 days). 

 Generous incision and debridement of all necrotic tissues form the cornerstone of surgical management. This had to be repeated several times in many patients to completely remove all the necrotic tissue. The mean number of surgical debridement employed in our patients was 2.

 Hyperbaric oxygen has also been used as an adjunct to surgery and antibiotics. It acts as a bactericidal / bacteriostatic agent against anaerobic bacteria by increasing the formation of free O2 radicals. It also restores the bacterial killing capacity of leucocytes in hypoxic wound by increasing tissue O2 tension. Some authors have reported a reduction in mortality and morbidity in NG with the use of Hyperbaric Oxygen [**24**]. We did not have access to this facility.

### Prognosis 

Even with appropriate treatment, the mortality rate can be as high as 25% in NF. A 10% to 45% mortality rate is seen in cases of Fournier’s gangrene. Advanced age, two or more associated comorbidities and a delay in surgery for more than twenty-four hours adversely affected the outcome [**1**]. There is also considerable postoperative morbidity, as many patients have to undergo repeated extensive debridement including scarring and disfigurement after thin split skin grafts.

**Fig. 1:**
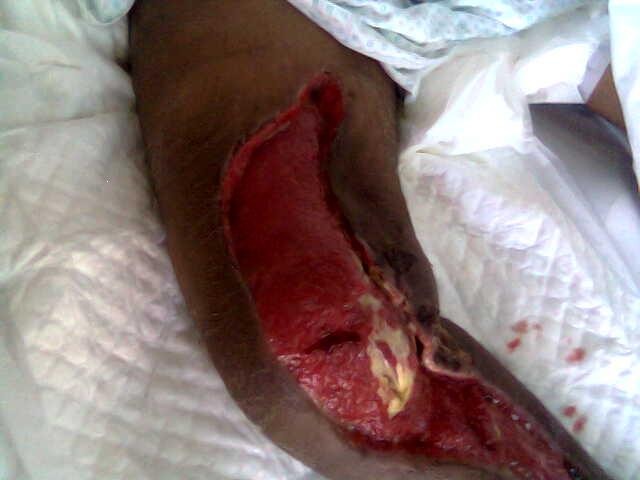
Healing wound after extensive debridement

**Fig. 2:**
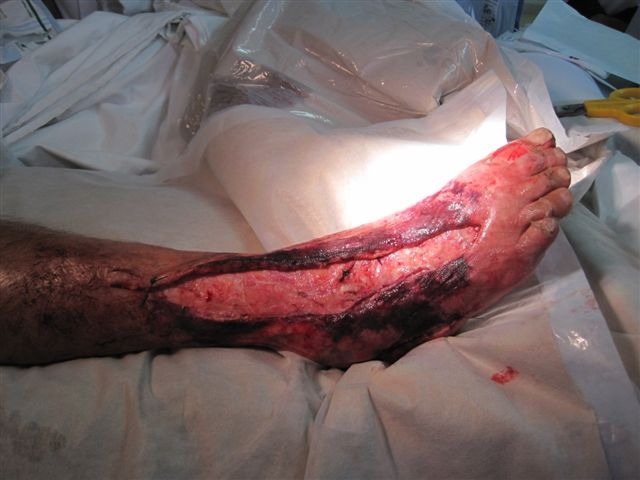
Extensive debridement of fascia. Unhealthy but viable skin is left

 Early recognition and expeditious initial wide excision and debridement along with appropriate antibiotic coverage and support of systemic effects of necrotizing fasciitis serve to decrease morbidity and mortality rates.

 Necrotizing fasciitis in children presents several dilemmas. First, there is the dilemma of diagnosis; the more often the injury is minor, the patients do not appear febrile or ill, laboratory findings may be initially normal and cultures may be negative. The second dilemma is how much tissue to debride, the selection of adjunct therapies and the way of combining reconstruction techniques for the best cosmetic result in the shortest time. The third dilemma is how to assemble a multidisciplinary team to meet the unique emotional, social, and medical needs of children. Since this case, we have developed a limb salvage team and a comprehensive order, as it is critical that emergency departments quickly recognize necrotizing fasciitis and initiate a rapid, multidisciplinary treatment plan. Despite the challenges of treating pediatric necrotizing fasciitis, a successful outcome can be achieved with prompt multidisciplinary management and a creative approach to reconstruction [**30**].

## Conclusion

 Necrotizing fasciitis is a rare but potentially fatal disease. It is a surgical emergency with a high morbidity and mortality rate. This condition is more common in males, diabetes mellitus being the most common comorbid disease. A high index of suspicion is called for in early diagnosis of this condition due to the paucity of specific cutaneous findings.

KEY POINTS 

 • Necrotizing fasciitis is a rare but potentially fatal disease; it can occur in all parts of the body, including the oral cavity and the perineum.

• Typically, patients who develop type 1 (polymicrobial) necrotizing fasciitis are immunocompromised in some way. However, with the type 2 (monomicrobial) variety, patients are usually immunocompetent with a history of trauma (sometimes minor).

 • Diagnosis is often delayed because of a paucity of signs and a low index of suspicion.

 • Necrotizing fasciitis is a clinical diagnosis. Blood tests and imaging, especially magnetic resonance imaging and computed tomography scans, can be helpful but are not diagnostic. Surgical exploration is advised if clinical suspicion is high.

 • The management includes fluid resuscitation, if indicated, intravenous broad-spectrum antibiotics, and early surgical debridement. Increasingly, community-acquired methicillin-resistant Staphylococcus aureus is being identified as the infective agent.
